# Confident gene activity prediction based on single histone modification H2BK5ac in human cell lines

**DOI:** 10.1186/s12859-016-1418-6

**Published:** 2017-01-25

**Authors:** Fereshteh Chitsazian, Mehdi Sadeghi, Elahe Elahi

**Affiliations:** 1 Institute of Biochemistry and Biophysics, University of Tehran, Tehran, Iran; 2 National Institute for Genetic Engineering and Biotechnology, Tehran, Iran; 3 School of Biology, College of Science, University of Tehran, Tehran, Iran; 4 Department of Biotechnology, College of Science, University of Tehran, Tehran, Iran

**Keywords:** Histone modifications, Transcription, CART, MDR, H2BK5ac

## Abstract

**Background:**

The histones in the core of nucleosomes may be subject to covalent post-transcriptional modifications. These modifications are thought to correlate with and possibly affect various genomic functions, including transcription. Each modification may alone or in combination with other modifications influence or be influenced by transcription. We aimed to identify correlations between single modifications or combinations of modifications at specific nucleosome sized gene regions with transcription activity based on global histone modification and transcription data of human CD4+ T cells and three other human cell lines. Transcription activity was defined in a binary fashion as either on or off. The analysis was done using the Classification and Regression Tree (CART) data mining protocol, and the Multifactorial Dimensionality Reduction (MDR) method was performed to confirm the CART results. These powerful methods have not previously been used for analysis of histone modification data.

**Results:**

We showed that analysis of the single histone modification H2BK5ac at only four gene regions correctly predicted transcription activity status of over 75% of genes in CD4+ T-cells. The H2BK5ac modification status also had high power for prediction of gene transcription activity in the three other cell lines studied. The informative gene regions with the H2BK5ac modification were all positioned proximal to transcription initiation sites. The CART and MDR methods were appropriate tools for the analysis performed. In the study, we also developed a non-arbitrary protocol for binary classification of genes as transcriptionally active or inactive.

**Conclusions:**

The importance of H2BK5ac modification with regards to transcription control has not previously been emphasized. Analysis of this single modification at only four nucleosome sized gene regions, all of which are at or proximal to transcription initiation, has high power for prediction of gene transcription activity.

**Electronic supplementary material:**

The online version of this article (doi:10.1186/s12859-016-1418-6) contains supplementary material, which is available to authorized users.

## Background

Gene functions of eukaryotic genomes including DNA replication, DNA repair, recombination, and transcription occur in the context of chromatin. Nucleosomes are the units of the primary level of chromatin structure. An octamer that contains two of each of the four histones H2A, H2B, H3, and H4 constitutes the protein core of each nucleosome, and its nucleic acid component consists of 147 bp of dsDNA wrapped around the surface of the protein core. Consecutive nucleosomes are separated approximately by 60 bp of DNA [1–4]. The nucleosome histones are subject to covalent post-translational modifications, the most frequent of which are methylations and acetylations [5–7]. The modifications generally occur at specific amino acid residues within the “tail” domains of the histones which are positioned at the N-terminus of the proteins and which are exposed and are protease sensitive, suggesting availability for interactions with other proteins [8–10]. The histone modifications may affect genomic functions by inducing changes in chromatin structure or by influencing interactions with proteins involved in genomic functions [11, 12]. The term “histone code” emphasizes the proposal that the modifications have important biological consequences [13–15]. The letters of the code include the multitude of known histone modifications and the positions of respective nucleosomes with relation to genomic regions of interest. Clearly, the code may be complex and may make use of several modifications at multiple positions. Among the various genomic functions, the potential consequences on transcription have been studied most widely [5, 16–18]. Here too, we aimed to identify the most informative correspondence between pattern of histone modifications and transcription using the Classification and Regression Tree)CART( [19] and the exhaustive Multifactorial Dimensionality Reduction (MDR) methods [20, 21]. Unlike some earlier studies wherein transcription levels were studied on a continuous scale, gene transcription activity in this study was considered as a binary property of being on (active) or off (inactive) [22]. In order to classify genes as active or inactive, a protocol for designating a value threshold was developed.

While CART and MDR are both combinatorial based data mining methods designed to handle large volumes of data, neither has been used previously to establish a relationship between histone modification patterns and gene transcription activity. CART and MDR both create novel informative attributes based on data from multiple discrete attributes. CART uses a tree-based algorithm to create a tree in which each node splits into two offspring nodes based on parameters under investigation and a binary response to a question; the more closely nodes are positioned to the root of the tree, the more important are their effects [23]. Here, the parameters under investigation were histone modifications at specific positions of genes and the question is whether a gene is active or inactive. The tree-based algorithm dictates that modification combinations created at new nodes necessarily include all upper level modifications; this feature of CART precludes consideration of all potential combinations of histone modifications. MDR uses a non-parametric algorithm wherein correlations between all possible combinations of modifications and gene activity status are considered when assessing the most informative combination of a specified number of modifications [23–25]. Compared to CART, MDR is a more thorough protocol, but also more time consuming. Genome wide histone modifications and transcription data from human CD4+ T-cells were investigated in a two-step study protocol. First, we aimed to identify interactions among histone modifications that were potentially informative with respect to gene activity using a portion of the CD4+ T-cell data. Secondly, having predicted potentially meaningful interactions, we went on to test their accuracy rate for predicting gene activity status. Finally, the results obtained data from CD4 + T- cells were applied to three other human cell lines.

Our findings suggest that data on two modifications at four nucleosome sized gene regions provide maximum power for prediction of gene activity in CD4+ T- cells, and that knowledge of presence or absence of the single modification H2BK5ac at four gene regions reveals almost the same amount of information. Taking into account results on all cell lines studied, the potential significance of H2BK5ac at various transcription start proximal sites is suggested. These findings beg additional research to learn how H2BK5ac may exert its effects. Additionally, it has empirical significance because it allows prediction on whether a gene is transcribed or not transcribed by analysis of status of a single type of histone modification. To the best of our knowledge, this option was not evidenced in results of earlier studies on relation between histone modifications and gene transcription [22].

## Methods

Genome-wide maps of histone modifications in human CD4+ T-cells, which to the best of our knowledge represent the most thorough analysis of histone modifications in human cells, were used in this study. The maps were obtained using the chromatin immunoprecipitation-sequencing protocol (ChIP-Seq) [26, 27]. Antibodies used for histone modifications were prepared in rabbits. The modifications analysed included 20 methylations and 18 acetylations. Presence of the histone variant H2AZ was also considered and was regarded the 39^th^ modification. The data were downloaded from the Human Histone Modification Database (HHMD; http://202.97.205.78/hhmd/Download.jsp) [28]. For data presentation, the genome was divided into 200 bp bins, each bin considered a single genomic region; ChIP signals (i.e. tag counts) for each of the modifications in each of the bins were recorded. These data were transformed into an m × n matrix (MATRIX 1), where m is the index of genomic regions and n is constituted by the 39 histone modifications. Cells within the matrix were occupied by 0 or 1 depending, respectively, on whether the value of the reported ChIP-seq signal pertaining to a designated modification in a designated gene region was, 0 or a value >0. For all modifications, the reported ChIP signals at various gene regions was usually either 0 or 1, but occasionally values of several hundred were also reported. Because the very rare positions with very high signals in even one gene would skew and confound our results, we resorted to using the binary status of presence or absence of modification. This was represented in the matrix, respectively, by values of 1 or 0. Additionally, ChIP-seq data for chromatin of CD4+ T-cells using non-specific goat (GEO: GSM393954) and rabbit IgG (GEO: GSM393955) antibodies were mapped to the chromosomes [29]. The sequence tags obtained with this data served as control. Gene expression data from 78 human CD4+ T-cell samples from 14 studies (GEO: GSE9927 [30], GSE14789 [31], GSE12079 [32], GSE16461 [33], GSE14278 [34], GSE25087 [35], GSE14924 [36], GSE6338 [37], GSE22045 [38], GSE26928 [38], GSE28490 [39], GSE28491 [39], GSE28726 [39], and GSE31773 [40]) were obtained from the Gene Expression Omnibus site (GEO; http://www.ncbi.nlm.nih.gov/geo/). Only data from normal untreated cells were downloaded. All the data were derived using the Affymetrix Gene Chip GPL570 platform which addresses 18,729 genes (Affymetrix Human Genome U133 Plus 2.0 Array; Affymetrix, Santa Clara, CA). Gene expression data from each file were normalized by application of the Robust Multi-array Average(RMA) algorithm, and normalized signals for each gene on the 78 files were averaged [41]. Pearson’s correlation coefficients for the 78 datasets were calculated.

### Frequency of various histone modifications at transcription initiation and termination sites of genes with different transcription levels

Initially, the 18,729 genes queried on the expression chips were grouped into 19 consecutive groups based on the averaged transcription value levels of the genes; each group contained 985 or 986 genes. The number of gene groups (19) represents a broad range of transcription levels. All 39 histone modifications were analysed. Consecutive 200 bp regions along the length of each chromosome were considered genomic regions. Transcription start site (TSS) and transcription termination site (TTS) for each of the 18,729 genes were defined according to Build GRCh36.3 of the human genome sequence (https://www.ncbi.nlm.nih.gov/). The 200 bp region containing the TSS and TTS sites of each gene were designated, respectively, the TSS and TTS gene region of the gene. Presence or absence of the modifications in five localities that together are expected to encompass 24 gene regions were considered for each gene: transcription start site (TSS), 2000 bp (10 gene regions) upstream of TSS (TSS-1 to TSS-10), 400 bp downstream of TSS (TSS + 1 and TSS + 2), site of transcription termination (TTS), and 2000 bp (10 gene regions) downstream of endpoint of transcription (TTS + 1 – TTS + 10) (Additional file 1: Figure S1). For each histone modification, a graph was plotted to show its occurrence frequency at the transcription start site (TSS) of genes within each of the 19 group of genes. Similar graphs were plotted for the transcription termination site (TTS). Data obtained from non-specific goat and rabbit antibodies were also plotted. Occurrence frequencies of modifications at TSS and TTS rather than at multiple or all positions simultaneously were considered because it was possible that unequal distribution of occurrence frequencies at different positions would cancel each other and thus preclude identification of an informative modification at a particular gene region. TSS and TTS gene regions were analysed because they are landmarks of the transcription process. The occurrence frequency of each modification for each group (F_g_; g = 1–19) was defined as the number of occurrences of that modification in the genes of that group (N_g_; g = 1–19) divided by number of occurrences of the modification in all genes of all groups (N_all_).”

1 $$ {F}_g=\frac{N_{\mathit{\mathsf{g}}}}{N_{all}} $$

### Identification of correlations between histone modification patterns and transcription of genes using CART and MDR

Analysis with CART and MDR were performed, respectively, using MATLAB and publicly available software (http://www.multifactordimensionalityreduction.org/) [42]. Using data from MATRIX 1, a new 936 by 6000 matrix (MATRIX 2) was prepared that included the (0/1) status of each of the 39 histone modifications at each of the 24 nucleosomes (39 × 24 = 936) of each of 6000 genes (Additional fie 1: Figure S1). The 6000 genes included the 3000 with the highest averaged expression value and the 3000 with the lowest. The former and latter, respectively, were considered active and inactive genes; gene activity was thus considered a discrete parameter. Based on empirical data on 43 human tissues, at least 36% and at most 55% of human genes are expressed in any tissue [43]. 3000 (~16% of 18,729 genes queried on chips) was thus considered a conservative number of genes expressed in any one cell type. Selection of a yet smaller number of genes may have limited our analysis to only very highly expressed genes. A table that listed the 6000 genes and their activity status (active/inactive) was prepared, and submitted to CART along with MATRIX 2. Matrices based on non-specific goat and rabbit antibody data were also submitted. CART produced trees with variable number of nodes. CART analyses with the 6000 genes were done in order to establish a threshold value for classification of each of the 18,729 genes in the gene expression data as active or inactive as described below. Subsequently, 24 separate analyses, one for each of the gene regions, were performed. For each of these, data on the presence (1) or absence (0) of each of the 39 histone modifications at the respective gene region of all the 18,729 genes, along with the threshold based activity status of the genes were submitted to CART. Finally, an analysis similar to the one described for each of the single gene regions alone was performed using modification data on all 24 gene regions of half of the 18,729 genes (train set of genes), and results were used to predict activity of the remaining half (test set of genes). From these analyses, the most informative histone modification pattern at all candidate gene regions, as well as the pattern at each of the 24 regions that best distinguished active and inactive genes were derived. (See flow chart in Additional file 1: Figure S2). MDR analyses of each of the 24 gene regions of the 6000 genes that included the 3000 with the highest and the 3000 with the lowest expression levels were also performed. The results of these analyses were compared with the results of CART analyses. Finally, Support Vector Machine (SVM) analysis was performed using data pertaining to all 39 modifications at the gene regions of all 18,792 genes. SVM is an established supervised machine learning based protocol that can be used for data classification [44]. The details of the protocols used are clarified below.

### CART analysis in three additional human cell lines

Capacity of H2BK5ac to predict gene activity in a binary fashion was tested on data from three additional human cell lines: IMR-90 (a fetal lung fibroblast cell line), hESC-h1 (an embryonic stem cell line), and MSC (a mesenchymal stem cell line) (Additional file 1: Table S1). These cell lines were chosen because genome wide histone modification data that included H2BK5ac and genome wide expression data were available for them. Analysis protocols similar to those used for CD4+ T-cells were used. Briefly, the ChromHMM binary package was applied to achieve a 0/1 binary classification for each of the histone modifications. The raw histone modification data for these cell lines did not contain a predominance of zero values. ChromHMM is an automated computational system based on a multivariant hidden Markov model that was developed for learning chromatin states and characterizing their biological functions [45]. Subsequently, a threshold for classification of active genes was derived for each cell line using expression data on 1/6 of genes with highest expression values and 1/6 of genes with lowest expression values. The threshold was used for classification of all genes as either active or inactive. Finally, status of the histone modifications on the 24 gene regions of 50% of genes chosen randomly (train set of genes) along with their threshold based predicted gene activity status were submitted to CART. The tree derived was used to predict gene activity status of the remaining 50% of genes (test set of genes). Prediction accuracy frequency was defined as the percent of correspondence between gene activity status based on threshold values and gene activity status based on the derived CART tree.

## Results

### Correlation between the 78 CD4+ T-cell gene expression data

The gene expression data from the 78 human CD4+ T-cell samples correlated quite well, thus justifying use of averaged normalized values. Pearson’s correlation coefficient for 90.64% of the comparisons was higher than 0.80, and 0.63 was the lowest correlation coefficient obtained. The *P* value for the correlations was <0.0001.

### Frequency of various histone modifications at transcription initiation and termination sites of genes with different transcription levels

**Fig. 1:**
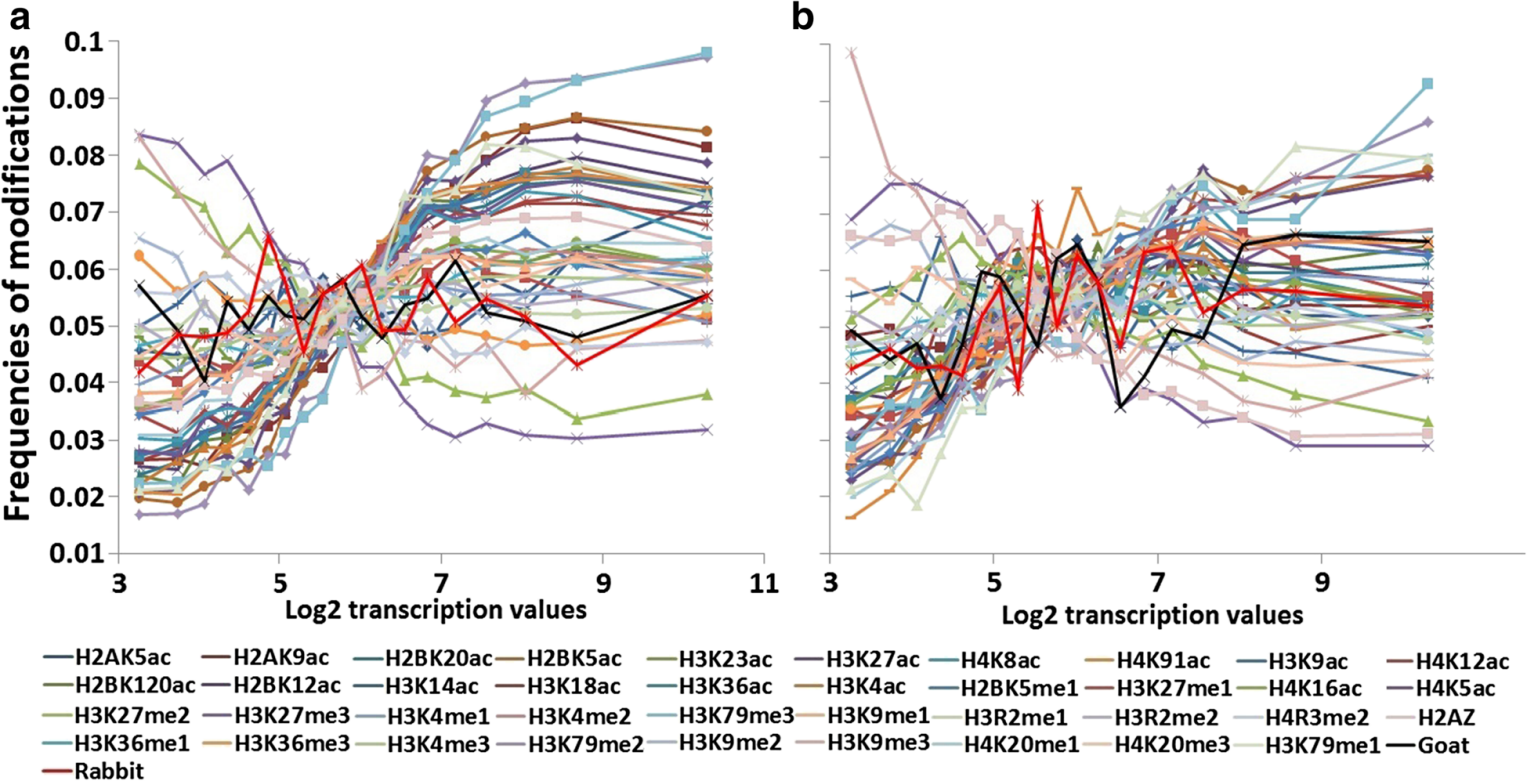
Frequencies of histone modifications at TSS and TTS of genes with different transcription levels. **a** TSS **b** TTS. Each curve is defined by 19 points, one for each of 19 gene groups; each group consists of approximately 1000 genes grouped on the basis of levels of transcription values. The 39 histone modifications are distinguished by color. Data on control nonspecific goat and rabbit antibodies are also presented

The distribution of frequencies of various histone modifications at the TSS (Transcription Start Site) and TTS (Transcription Termination site) in the 19 groups of genes grouped on the basis of log2 levels of gene transcription value are shown in Fig. 1a, b. In Additional file 1: Figure S3, the distributions for each of the modifications at the TSS and TTS are shown separately. This preliminary analysis was performed in order to detect an obvious correlation that may exist between a specific histone modification and gene activity. An overall comparison of curves for TSS and TTS in Fig. 1 suggests greater variation in the frequencies of various acetylation modifications at TSS among gene groups with different levels of expression. As compared to TSS curves, TTS curves tend to have shallower slopes and often approach a horizontal line. At least 27 modifications at TSS, including all acetylations except H3K14ac, were associated with increased gene activity. Only three modifications at TSS showed association with decreased activity. The distribution of frequencies of nine modifications at TSS showed little or no correlation with gene activity. From Fig. 1 and Additional file 1: Figure S3, it is evident that H3K79me3, H3K79me2, H2BK5ac, H3K27me2, H3K27me3, and H3K9me3 at TSS are among the modifications with frequencies best associated with levels of transcription. The occurrence frequencies of the first three modifications were relatively high in groups of genes with relatively higher levels of expression, and declined in groups with lower levels of expression. H3K27me2, H3K27me3, and H3K9me3 showed the opposite pattern. These results were the first indication that H2BK5ac is among the modifications strongly associated with gene activity levels. The various histone modification curves nearly converged at a frequency that was close to the random expected frequency (1/19 = 0.053) (Fig. 1). The convergence is more blurred at TTS as compared to TSS. The point of convergence can be considered a transition point. Clearly, this transition point is most biologically informative for those modifications that crossed it with the largest slope; these are the modifications identified above. With respect to gene activity, the gene groups were distributed approximately equally to the left and right of the transition point.

### Establishment of criterion for assessment of active genes and classification of the 18,729 genes as active or inactive

**Fig. 2:**
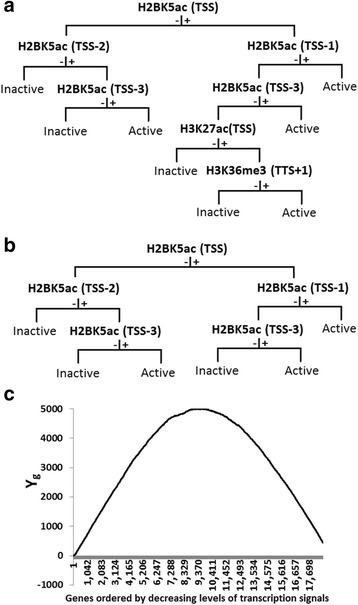
Use of CART tree based on 6000 genes for assessment of active/inactive status of genes. **a** and **b** Respectively, the seven and five node trees generated by CART based on histone modification patterns of the 3000 genes with highest transcription values (designated active) and the 3000 with the lowest values (designated inactive). + and -, respectively, signify presence or absence of modification in preceding node. See Additional file 1: Figure S4. **c** Plot showing pattern of Y_g_ values of 18,729 genes ordered from left to right by decreasing levels of transcription values

One to seven node CART (Classification and Regression Tree) trees for distinguishing between histone modifications patterns of the 3000 most active and 3000 most inactive genes were generated. When the histone modification patterns on the 24 gene regions was substituted by goat and rabbit immunoglobulin binding data, CART was unable to generate a seven node tree or smaller trees with a *P* value of <0.05. The 18,729 genes were then ordered in sequence of decreasing averaged normalized expression values, and each gene was given an index of +1 or -1 based, respectively, on presence or absence of the histone modification pattern generated in each of the seven CART trees. In other words, the designations were performed seven times. To establish the most parsimonious histone modification tree to be used for further analysis, two by two correlations between +1/-1 designations of the 18,729 genes using trees with the various numbers of nodes were calculated (Table 1). The correlation values between results of five node and four node trees, six and five node trees, and seven and six node trees were, respectively, 0.85, 0.95, and 0.98 (Table 1). It was considered that the five node tree contained the bulk of information with respect to relation between histone modification patterns and gene transcription levels, and that addition of a sixth node would contribute minimally to this information. The seven and five node trees generated by CART based on data on the 6000 genes are presented in Fig. 2a and b.

**Table 1:** Correlations between active/inactive designations of genes based on CART trees^a^ with increasing number of nodes

No. nodes in tree 1	No. nodes in tree 2	Correlation^b^
1	2	0.8
2	3	0.81
3	4	0.82
4	5	0.85
5	6	0.95
6	7	0.98

^a^Based on 3000 genes with highest transcription values and 3000 genes with lowest values. ^b^Pearson’ s correlation

The +1/-1 designations of the 18,729 genes based on the five node tree were now used to establish a threshold transcription value for classification of the genes as either active or inactive. For this purpose, a value Y was calculated for each gene (Y(g)), where g is the position of the respective gene in the list of genes ordered on basis of transcription value and where,

2 $$ Y(g)={\displaystyle {\sum}_{\mathrm{i}=1}^g\kern0.5em \left({\mathrm{x}}_{\mathrm{i}}\right);{\mathrm{x}}_{\mathrm{i}}=+1\kern0.5em \mathrm{or}-1.} $$

The pattern of Y values for the 18,729 genes based on the five node tree is shown in Fig. 2c. The position (g_max_) in the ordered list of genes of the gene associated with the highest Y value was identified using the following formula:

3 $$ {\mathrm{g}}_{\max } = \mathrm{argmaxY}\left(\mathrm{g}\right);\mathrm{n} = 18729 $$

$$ \mathrm{g} = 1, \dots,\ \mathrm{n} $$

Y_max_ associated with the gene at g_max_ was 4977; log_2_ of the corresponding value (5.92) was designated the threshold for considering genes as active. The gene associated with the Y_max_ value and all genes at higher positions in the list of genes were considered active, and the genes at lower positions were considered inactive. An alternate calculation identified the same threshold level. Consider the frequency of +1 values among all 18,729 genes (= +1_all_), among all the genes above each of the genes in the list (= +1_g-above_), and among all the genes below each of the genes in the list (= +1_g-below_). For each gene position, a comparison is made between (+1_g-above_) and (+1_all_) and also between (+1_g-below_) and (+1_all_), and *P* values are calculated. The log_2_ of the transcription value of the gene wherein (P_+1g-above:+1all_ + P_+1g-below:+1all_) reached a minimum (*P* <0.0001) corresponded to the threshold for active genes described above (i.e. 5.92). 8805 of the genes (47.01%) were classified as active and the remainder (9924 genes; 52.99%) were considered inactive. 6891 (78.26%) of the genes above the threshold level were in fact associated with index +1, and 7229 (72.84%) of the genes below the threshold level were in fact associated with index -1 (+1/-1 designations based on five node CART tree of Fig. 2b).

### Correlation between histone modifications at specific positions and gene transcription

The root of the seven node and five node CART trees based on the 3000 genes with highest and 3000 genes with lowest transcription values was occupied by a modification at the TSS (Fig. 2a, b). The next four nodes were occupied by modifications at TSS proximal positions TSS-1, TSS-2 and TSS-3. Notably, the modification at all five nodes of the five node tree was H2BK5ac. This finding suggests that presence of H2BK5ac may generally activate gene transcription. The significance of H2BK5ac at TSS for increased gene transcription had already been suggested by the analysis on distribution of the frequencies of the various histone modifications at this positions in 18,729 genes grouped on the basis of level of activity (Fig. 1a). In addition to the described modifications, the sixth and seventh nodes of the seven node tree suggest an activating influence for modifications H3K27ac at TSS and H3K36me3 at TTS+1 (Fig. 2a).

**Fig. 3:**
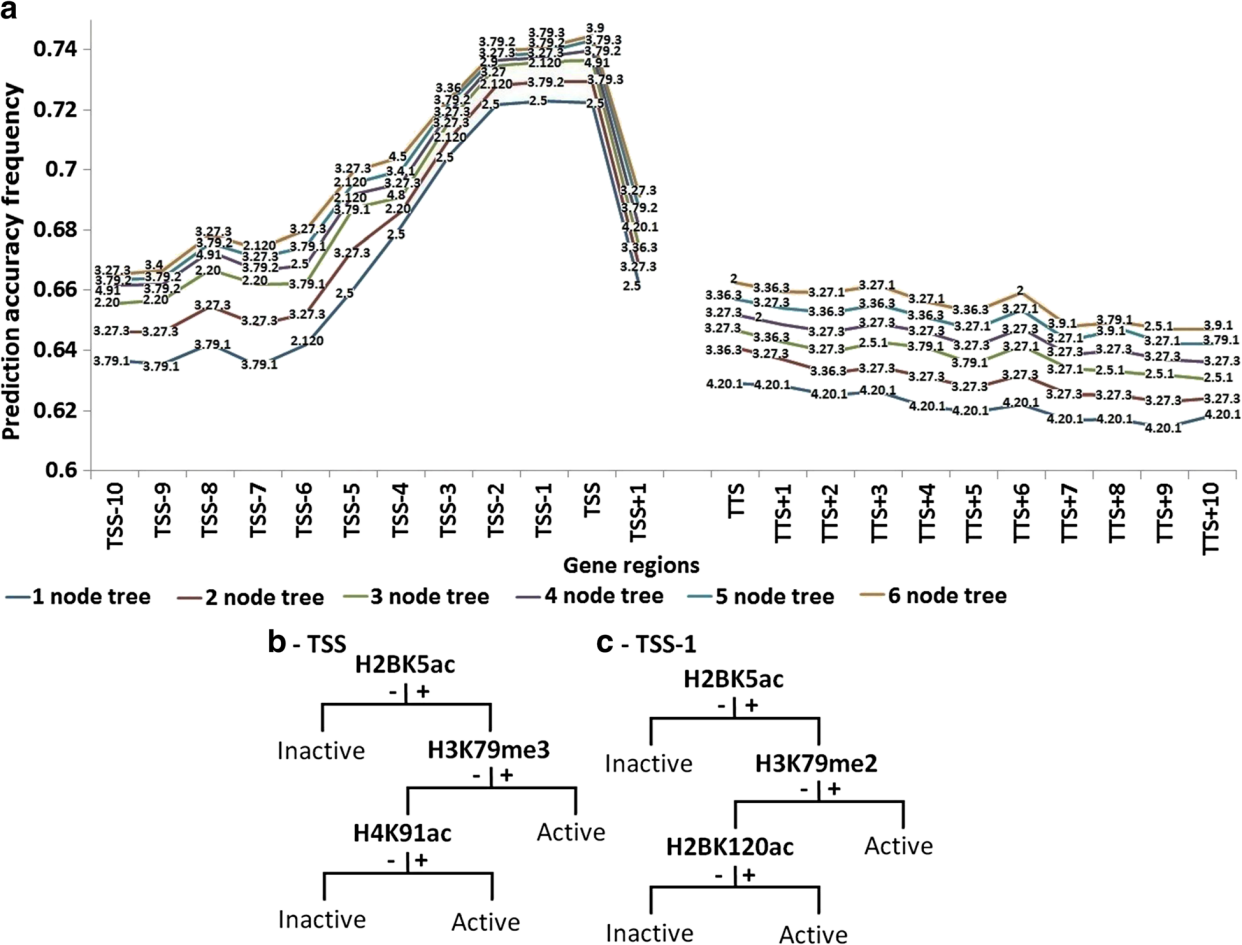
Gene activity predictions based on histone modification patterns at single gene regions for all 24 gene regions. **a** Histone modification patterns in CART trees generated using single gene region data were used to ascribe index values of +1 and -1 to each of the 18,729 genes, and the percent of genes with index values of +1 and -1 that were, respectively, among genes designated active and inactive based on Ymax threshold was calculated. The percent was considered the prediction accuracy frequency of the tree. The histone modifications at nodes of each gene region are shown with two (for acetylations) or three (for methylations) digits separated by dots. The first and second digits indicate histone number and the amino acid number. The third digit when present represents the number of methyl groups present. **b** and **c** Respectively, three node CART trees generated on basis of histone modification patterns of TSS and TSS-1 of 18,729 genes. + and -, respectively, signify presence or absence of modification in preceding node. See Additional file 1: Figure S4. Prediction accuracy frequency of modification patterns at TSS+2 (not shown) were much lower than those at TSS+1

It was considered that other histone modifications not evidenced in the described trees may also influence gene activity, and that their contributions are being masked by the dominant effects of the few modifications apparent in these trees. We attempted to achieve a more complete understanding of effects of other histone modifications on gene activity by analyzing modification patterns at specific gene regions. To this end, data showing the presence or absence of each of the 39 histone modifications at each of the 24 regions of all the 18,729 genes, along with the threshold (Y_max_) based activity status of the genes, were submitted to CART. CART generated 24 trees, one for each of the gene regions. Each tree was pruned to contain only the root node, and then successively larger number of nodes. Genes were assigned index values of +1 or -1, based on CART trees with variable numbers of nodes. The prediction accuracy frequency of each of the pruned trees for each of the gene regions was estimated based on per cent of genes whose index (+1 or -1) matched the activity status based on the Y_max_ threshold (Fig. 3a). Based on this parameter, the analysis revealed that the most informative sites are TSS, TSS-1, and TSS-2. There was an ostensible drop in prediction accuracy frequency for gene regions upstream of TSS-2 and downstream of TSS. Gene activity predictions using three, four, five, and six node trees based on modifications at either TSS, TSS-1, or TSS-2 were the same as predictions made based on Y_max_ for nearly 74% of the genes. The information content of other gene regions decreased the farther they were positioned from the TSS (the more upstream). The prediction accuracy frequency at all regions increased with addition of new nodes, but the increase was not notable after approximately three nodes. Another word, the information content at each gene region is largely contained within the status of only few modifications. Three node trees for TSS and TSS-1 are shown in Fig. 3b, c. H2BK5ac, H3K79me3, and H4K91ac constitute the three nodes in the TSS tree, and H2BK5ac, H3K79me2, and H2BK120ac constitute the nodes of the TSS-1 tree. H2BK5ac occupied the root of trees for all positions between TSS-5 and TSS+2. Once again, this observation indicates the importance of this modification. In fact, it appears that the absence of H2BK5ac at these positions promotes absence of gene activity. Prediction accuracy frequencies of modifications at TTS and various TTS proximal positions were similar and all were nominal as compared to TSS (Fig. 3a).

**Fig. 4:**
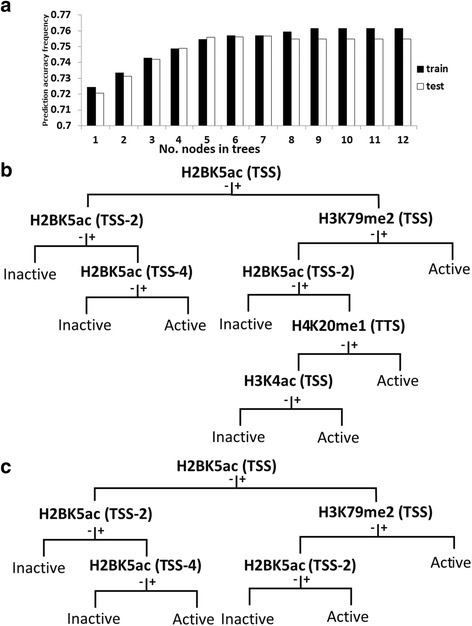
Gene activity predictions on test genes based on CART trees generated with train gene data. **a** Histone modification patterns in CART trees with various numbers of nodes were generated using data on all 24 gene regions of approximately 10,000 training genes, and the trees were used to ascribe index values of +1 and -1 to each of the same (left columns; training) or different (right columns; test) set of genes. The percent of genes with index values of +1 and -1 that were, respectively, among genes designated active and inactive based on Y_max_ threshold was calculated. The percent was considered the prediction accuracy frequency of the tree. **b** and **c** Respectively, seven and five node CART trees generated by using data on all 24 gene regions of approximately 10,000 training genes. + and -, respectively, signify presence or absence of modification in preceding node. See Additional file 1: Figure S4

An analysis similar to that described for single gene regions was performed on all 24 gene regions of subgroups of the 18,729 genes. Here, the status of the 39 modifications for all the gene regions for half the 18,729 genes chosen at random (training genes), and the activity status of the same genes based on the Y_max_ threshold were submitted to CART. A very large tree was generated. The tree was pruned, and the prediction accuracy frequency of one to seven node trees was estimated exactly as described above for the single gene region analysis. The prediction accuracy frequency of the trees was assessed on the training genes as well as on the set of remaining genes (test genes), and maximum prediction powers of 75.70 and 75.68%, respectively, were achieved based on a seven node tree (Fig. 4a, b). The prediction accuracy frequency of the tree improved as more nodes were added, but only marginally after the fifth node. Its value for the test genes using the five node tree was 75.59%. The same results were obtained when a second group of genes was randomly selected to constitute the training genes. As before, H2BK5ac occurred frequently in the five node tree and comprised four of its five nodes (Fig. 4c). Notably, empirical results on gene expression in CD4+ T-cells were consistent with our findings; the experimental analysis classified 49.45% of the genes as active and 50.55% as inactive [43]. Furthermore, active/inactive classification by our bioinformatics approach (i.e. based on five node CART tree of Fig. 4c) and by the experimental approaches were the same for 89.94% of the genes (Additional file 1: Table S2).

**Fig. 5:**
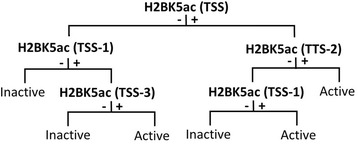
CART trees based on single modification H2BK5ac. Five node CART tree generated on basis of presence or absence of single modification H2BK5ac at all 24 gene regions of all 18,729 genes and activity status of the genes based on Y_max_ threshold is shown. + and -, respectively, signify presence or absence of modification in preceding node. See Additional file 1: Figure S4

Finally, having evidenced the significance of H2BK5ac, we submitted to CART only the status of this modification at all 24 gene regions of all the 18,729 genes and the threshold (Y_max_) based activity status of the genes. The five node tree generated had a prediction accuracy frequency of 75.33% on the same set of genes, which is very similar to the predictive accuracy frequency (75.59%) of the same sized tree generated using data on all 39 modifications (Fig. 5). Again, most of the power provided by the status of H2BK5ac was embedded in its status at TSS and TSS proximal positions. A two node tree revealing status of H2BK5ac at only the two positions TSS and TSS-2 had a notable prediction accuracy frequency of 72.30%. To get further assurance that H2BK5ac status at TSS proximal gene regions is more informative than the status of any other single histone modification, we generated separate five node CART trees for each of the remaining 38 modifications on the 24 gene regions. These trees correspond to the tree for H2BK5ac in Fig. 5. The prediction accuracy frequency of each tree was then calculated (Table 2). Results show that H2BK5ac is indeed the best predictor of gene activity status in in CD4+ T-cells. The next best predictor is H4K91ac.

**Table 2:** Gene activity prediction frequencies for CD4+ T cells based on five node CART trees for each of 39 histone modifications on the 24 nucleosome sized regions

Histone modification	H2BK5ac	H4K91ac	H3K9ac	H2BK120ac	H3K27ac	H3K4ac	H3K79me3	H3K79me2	H4K5ac	H3K36ac
Prediction accuracy	0.753	0.723	0.720	0.718	0.713	0.713	0.711	0.707	0.701	0.695
Histone modification	H4K8ac	H3K18ac	H2BK20ac	H2AZ	H3K79me1	H2AK9ac	H3K4me2	H4K20me1	H4K16ac	H4K12ac
Prediction accuracy	0.693	0.687	0.681	0.674	0.670	0.666	0.662	0.661	0.653	0.645
Histone modification	H3K9me1	H3K4me3	H3K36me3	H3K27me1	H2BK5me1	H3K27me3	H3K4me1	H2AK5ac	H3K9me3	H3K23ac
Prediction accuracy	0.644	0.642	0.637	0.633	0.629	0.627	0.610	0.603	0.600	0.594
Histone modification	H3K27me2	H3K36me1	H3K14ac	H3K9me2	H4K20me3	H4R3me2	H3R2me1	H3R2me2	H2BK12ac	
Prediction accuracy	0.572	0.566	0.543	0.542	0.537	0.531	0.531	0.524	0.486	

### MDR (Multifactorial Dimensionality Reduction) analysis

Because the CART algorithm precludes consideration of all potential combinations of histone modifications, it was possible that the most informative multi-attribute pattern of histone modification was not recognized while using the CART software. With this consideration, analysis was also performed with MDR. At first, histone modifications at each of the 24 gene regions of the 3000 genes with highest values and 3000 genes with lowest values (respectively, active and inactive genes) were analyzed separately by the MDR software. The software identified the single modification at each of the gene regions that had greatest difference in frequency between the active and inactive group of genes. In addition to single modifications, combinations of modifications with up to five modifications with greatest difference in frequency were also identified (Table 3). Results of the MDR analysis compared well with results of CART analysis. Both identified TSS (score = 0.792) and TSS-1(score = 0.790) as the most informative positions, and H2BK5ac as the most telling single modification at both these positions. In fact, like CART, MDR reported H2BK5ac as the most informative modification at all gene regions from TSS-4 to TSS. The correspondences between the MDR and CART analyses are reassuring, particularly because the MDR analyses were independent of a calculated threshold for designation of genes as active. When considering all 24 gene regions, on the average 77.78 and 72.22%, respectively, of the one and four most informative modifications predicted by CART and MDR were the same. Considering that 39 modifications are being considered at each position, this degree of overlap is notable and suggests that truly informative modifications are being predicted by both programs.

**Table 3:** Comparison of MDR and CART assessments of combinations of histone modifications for correlation with transcription

Region	MDR: single^α^	Tba^b^	Root^c^	Identity^d^	MDR: 5 modifications^e^	Tba^b^	5 node tree^f^	Identity^d^
TSS-10	H3K79me1	0.6642	H3K79me1	+	2.20,4.91,3.27.3,3.79.1	0.719	3.27.3,2.20,3.79.1,4.91	100%
TSS-9	H3K79me1	0.6634	H3K79me1	+	2.20,3.27.3,3.79.2,3.79.1	0.7233	2.20,3.27.3,4.91,3.79.1	75%
TSS-8	H3K79me1	0.6717	H3K79me1	+	4.91,3.27.3,3.79.2,3.79.1	0.7327	3.27.3,2.20,3.79.1,4.91	75%
TSS-7	H2BK20ac	0.6686	H3K79me1	-	2.20,3.27.3,3.79.2,3.79.1	0.7368	3.27.3,2.20,3.79.1,3.79.2	100%
TSS-6	H2BK20ac	0.6856	H2BK120ac	-	2.20,2.5,3.27.3,3.79.1	0.7427	3.27.3,2.5,2.120,3.79.1	75%
TSS-5	H2BK120ac	0.7078	H2BK5ac	-	2.5,2.120,3.27.3,3.79.1	0.7638	3.27.3,3.79.1,2.120,2.5	100%
TSS-4	H2BK5ac	0.7252	H2BK5ac	+	2.5,4.91,3.27.3,3.79.1,	0.7761	2.5,3.27.3,2.20,4.8	50%
TSS-3	H2BK5ac	0.7673	H2BK5ac	+	2.5,2.120,3.79.1,3.79.2	0.7945	2.5,3.27.3,2.120,3.27.3	50%
TSS-2	H2BK5ac	0.7893	H2BK5ac	+	2.5,2.120,3.27,3.27.3	0.8093	2.5,2.120,3.27,2.9	75%
TSS-1	H2BK5ac	0.7903	H2BK5ac	+	2.5,3.27,4.91,3.79.2	0.8125	2.5,3.27.3,3.79.2,2.120	50%
TSS	H2BK5ac	0.7923	H2BK5ac	+	2.5,4.91,3.79.2,3.79.3	0.8181	2.5,3.79.2,4.91,3.79.3	100%
TSS+1	H4K91ac	0.6963	H2BK5ac	-	2.5,4.91,4.20.1,3.79.2	0.7322	2.5,3.27.3,3.36.3,4.20.1	50%
TTS	H4K20me1	0.6731	H4K20me1	+	3.27.1,3.27.3,3.36.3,4.20.1	0.7238	3.27.3,4.20.1,3.36.3,3.27.3	75%
TTS+1	H4K20me1	0.6757	H4K20me1	+	H2AZ,3.27.3,3.36.3,4.20.1	0.7239	H2AZ,3.27.3,4.20.1,3.36.3	100%
TTS+2	H4K20me1	0.6677	H4K20me1	+	4.91,3.27.3,2.5.1,4.20.1	0.6986	3.27.3,4.20.1,3.36.3,3.27.3	50%
TTS+3	H4K20me1	0.6673	H4K20me1	+	3.27.1,3.27.3,3.36.3,4.20.1	0.7133	2.5.1,3.27.3,4.20.1,3.27.3	50%
TTS+4	H4K20me1	0.6635	H4K20me1	+	3.27.1,3.36.3,3.27.3,4.20.1	0.7084	3.27.3,4.20.1,3.79.1,3.27.3	50%
TTS+5	H4K20me1	0.6617	H4K20me1	+	2.5.1,3.27.3,4.20.1,3.79.1	0.7088	3.27.3,4.20.1,3.79.1,3.27.3	75%

^α^, MDR most informative single modification, ^b^: Testing bal. accuracy ^c^: Root of 5 node CART tree based on data presented in Fig. 3a, ^d^: Identity between MDR & CART, ^e^MDR: most informative combination of 5 modifications that are designated as described in legend to Fig. 3, ^f^: Modifications in 5 node CART tree based on data presented in Fig. 3a

### SVM (Support Vector Machine) analysis

Data pertaining to the 39 histone modifications at each of the 24 nucleosomes of each of 3000 genes with the highest averaged expression value and the 3000 with the lowest averaged expression value constituted the training data used by SVM. It is to be emphasized that as with the MDR analysis, a threshold for designating genes as active or inactive was not designated for the SVM analysis. One, two, and three component analyses were performed. The best results of all three analyses included H2BK5ac at TSS proximal gene regions (Table 4). Subsequently, results of the component analyses were used to predict gene activity status of all 18,792 genes and the predictions were compared with the threshold (Y_max_) based activity status of these genes. Correspondence between activity statuses is designated by an accuracy value by SVM. Accuracies of the best SVM component sets ranged from 0.72 to 0.75.

**Table 4:** Results of SVM analyses

No. components in analysis	Best component(s)	Accuracy
One	H2BK5ac at TSS	0.72
Two	H2BK5ac at TSS-1	0.74
	H2BK5ac at TSS-2	
Three	H2BK5ac at TSS-1	0.75
	H2BK5ac at TSS-2	

### CART analysis in three additional human cell lines

**Fig. 6:**
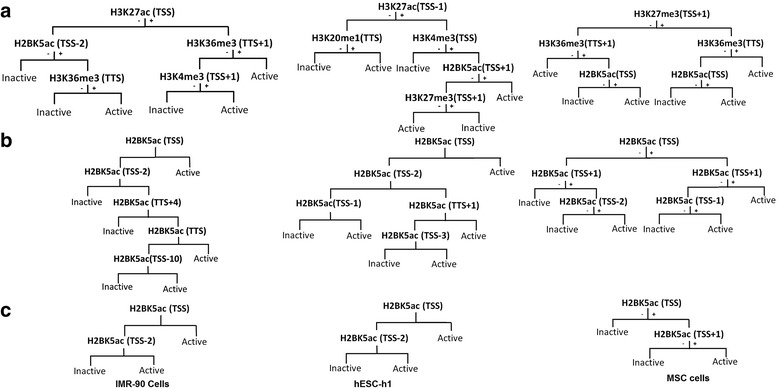
CART trees based on data from IMR-90, hESC-h1, and MSC cells. **a** Five node trees based on data from all histone modificatios. **b** Five node trees based only on H2BK5ac data. **c** Two node trees based only on H2BK5ac data. See Additional file 1: Figure S4

H2BK5ac occupied at least one node in each of the five node CART tree generated for IMR-90, hESC-h1, and MSC cell lines based on available histone modification data for the respective cell line (25 or 29 modifications; Additional file 1: Table S1) (Fig. 6a). The CART tree patterns observed suggest that H2BK5ac is an important factor with respect to gene activity in these cell lines, albeit less important than in CD4+ T cells. Gene activity prediction accuracy frequencies of the trees ranged from 68.3 to 78.69% (Table 5). To assess gene activity prediction capacity of H2BK5ac alone in IMR-90, hESC-h1, and MSC cells, two and five node CART trees based only on H2BK5ac data were derived (Fig. 6b, c). Optimal trees mostly included TSS proximal gene regions. The prediction accuracy frequencies of two node CART trees ranged from 63.83–78.10% (Table 5). These prediction accuracy frequencies, as compared to five node trees based on all histone modifications, were only 0.59 to 4.53% less.

**Table 5:** Gene activity prediction capacity in IMR90, hESC-h1, and MSC cells

Cell type	Two node CART tree based		Five node CART tree based		Five node CART tree based on	
	Gene regions at nodes	Prediction capacity	Gene regions at nodes	Prediction capacity	Modifications & gene regions at nodes	Prediction capacity
IMR-90 cells	TSS+1, TSS-2	78.10%	TSS+1, TSS-2, TTS+4, TTS, TSS-10	78.10%	H3K27ac/TSS, H2BK5ac/TSS-2 H3K36me3/TTS, H3K36me3/TTS+1, H3K4me3/TSS+1	78.69%
hESC-h1 cells	TSS, TSS-2	69.70%	TSS, TSS-2, TSS-1, TTS+1, TSS-3	70.00%	H3K27ac/TSS-1, H3K4me3/TSS, H2BK5ac/TSS+1, H3K27/TSS+1, H4K20me1/TSS	74.42%
MSC cells	TSS, TSS+1	63.83%	TSS, TSS+1, TSS-1	63.85%	H3K27me3/TSS+1, H3K36me3/TTS+1, H2BK5ac/TSS, H3K36me3/TTS, H2BK5ac/TSS	68.38%

## Discussion

The most important novel findings of this study are the potential significance of the histone modification H2BK5ac with relation to gene transcription, and the applicability of CART and MDR to analysis of histone modification data. Additionally, a non-arbitrary protocol for binary classification of genes as transcriptionally active or inactive was developed. In the analysis performed where gene activity was considered a binary parameter, it became evident that histone modification patterns that included only a few modifications at a few positions correctly predicted the activity status of approximately 75.59% of genes in CD4+T-cells. This prediction accuracy frequency is notable because a large number of genes are being considered. In fact, the probability of achieving this level of correct predictions simply by chance is < 0.0001, suggesting that the modification patterns identified are truly meaningful with respect to transcription activity. Clearly, transcription is affected by many parameters in addition to histone modifications, and it might be impossible to reach a prediction accuracy frequency much higher than that achieved here based only on histone modifications. Furthermore, it is to be emphasized that the notable prediction accuracy frequency based on a few histone modifications at a few positions does not necessarily indicate that higher dimensional combinations of histone modifications do not influence gene expressions. The effects of some modifications may be masked in the analyses performed if these putative modifications frequently co-existed with the modifications identified. In the analysis performed, only the modifications with the highest correlation with gene activity would be identified. In this study, we relied strongly on the CART program. This data mining method can consider simultaneously many parameters embedded in a large volume of data and, as compared to linear regression based methods, this is one of its advantages [22]. Nevertheless, CART is not an exhaustive protocol wherein all possibilities are considered. For this reason, to the extent possible, its results were compared with results obtained from the exhaustive MDR algorithm. MDR was able to perform analysis on all 39 histone modifications at any single gene region of 6000 genes. The high degree of correspondence between results of this analysis and the comparable analysis using CART, gives confidence that the more complex analyses performed only by CART provide reliable data. Similar to CART results, the MDR results highlighted the importance of H2BK5ac and of the TSS and TSS-1 positions. It was also reassuring that the machine learning based SVM analysis also signaled the significance of H2BK5ac at TSS and TSS proximal regions.

CART was initially used to establish a threshold for considering genes as either active or inactive. The validity of the threshold identified (corresponding to log_2_ of transcription value 5.9) is supported by three observations. One is the correspondence between MDR and CART results described above. Additionally, the threshold value is within the “transition region” identified by comparing frequencies of histone modifications of genes grouped on the basis of levels of transcription values (Fig. 1). This grouping and the MDR analysis were performed without consideration of a threshold for gene activity. Finally, there was correspondence between active/inactive gene classification based on empirical data and as predicted by CART (Additional file 1: Table S2) [43].

Notably, the ranking of frequencies of histone modifications in various groups of active genes (gene groups to the right of the transition point) are similar and the reverse rankings are observed for the inactive genes (gene groups to the left of the transition point) (Fig. 1). For example, H2BK5ac is among the most frequent modifications in all groups of active genes and, H3K27me3 is the most frequent modification in all groups of inactive genes. It is at the transition point that the pattern of histone modifications reverses, such that the more frequent on one side become the less frequent on the other side.

The five node CART tree generated on the basis of data on all histone modifications at 24 gene regions in a training set of approximately 10,000 genes has an activity prediction accuracy frequency of 75.59% (Fig. 4c). The tree is of practical significance not only because of its relatively high prediction capacity, but also because it only entails establishment of status of a single modification (H2BK5ac) at four positions, and the status of a second modification (H3K79me2) at a single position. The status of a single modification (H2BK5ac) at four gene regions, which can be established by using a single antibody, has nearly the same prediction accuracy frequency (75.33%; Fig. 5). These results are based on modification data on human CD4+ T-cells, but results derived from three additional human cell lines suggest that the effects and roles of H2BK5ac may be general. Although H2BK5ac was not the most informative histone modification in those cells, it consistently appeared at at least one of the nodes in the five node CART trees. Furthermore, its prediction accuracy capacity was very close to the capacity based on data on all histone modifications or capacity based on data on any single histone modification (Table 5, Additional file 1: Table S3). Consistent with findings presented here, it was reported that acetylation at H2BK5 is important for regulation of gene expression profiles in epithelial cells [46]. Among the various cell lines analyzed here, gene activity prediction accuracy based solely on H2BK5ac was highest for IMR-90 cells, then decrease progressively for CD4+ T-cells, hESC-h1 cells, and MSC cells (Table 5). The predictive capacity ranged from 70 to 78% for the first three cell lines which is impressive. The informative positions with the H2BK5ac modification in the different cell lines were mostly close to TSS. It may be that simply a concentration of H2BK5ac at TSS and few regions very close to TSS may be important for gene activity prediction.

As in the earliest studies on relation between histone modifications and gene transcription, we assessed that most modifications are associated with activation of genes, that all acetylations except H3K14ac have this effect, and that the associations between gene activity and methylations are more variable than the associations between gene activity and acetylations [6, 47–49]. The two most comprehensive previous analyses both relied on the whole genome histone modification data used in the present study [22, 26]. The first analysis was done by the group who produced the data [26]. A detailed comparison between effects of single modifications at the TSS assessed by us and by others at the 2 kb region surrounding the TSS revealed very similar results (compare Additional file 1: Figure S3 to Fig. 2c in reference [20]). In the earlier study, though not emphasized, H2BK5ac was in fact associated with increased gene activity more than any other single modification [26].

The objectives of the second study that used the histone modification data in CD4+ T cells were more similar to ours, in that in addition to single modifications, correlations between combinations of modifications and transcription activity were sought [22]. In that study, linear regression methods which allowed analysis of combinations of maximally three modifications were used. The authors identified H3K27ac as the most informative single modification, H3K27ac + H4K20me1 as the best modification pair, and H3K27ac + H3K4me1 + H4K20me1 as the best three modification combination. None of these modifications were included in the most informative five node CART tree we generated, although H4K20me1 was among the modifications the seven node CART tree (Fig. 4). The differences in results may be partly due to the fact that frequencies of modifications in a large 4001 bp region surrounding the TSS were analyzed in the earlier study, whereas our data are based on both nature of the modifications and specific gene regions. Our analysis included only two gene regions (TSS+1 and TSS+2, corresponding to 400 nucleotides) downstream of the TSS. The earlier approach could mask significant contributions of specific modifications at particular gene regions by an averaging effect. Notably, when the authors used combined data on the 142 most informative three modification combinations, H2BK5ac was identified as one of the four most important modifications [22].

We surmise that results of the two earlier studies that used CD4+ T-cell data signal the significance of the H2BK5ac modification. One of the earlier studies emphasized that a small number of modifications can predict gene expression accurately [22]. Our results suggest that a single modification, H2BK5ac, at only a few positions is highly informative.

## Conclusion

The most important finding with relation to gene transcription was the potential significance of the histone modification H2BK5ac at transcription start proximal sites. We showed that analysis of this single modification at only a few gene regions in CD4+ T-cells and three other cell lines has high power for prediction of gene transcription activity. The fact that assessment of only a single histone modification at only a few transcription proximal positions has high predictive power with respect to transcription is of practical importance.

## Additional file

Additional file 1: Figure S1. shows five localities that together are expected to encompass 24 gene regions. **Figure S2.** Flow chart of analyses performed with CART. **Figure S3.** Occurrence frequencies of individual histone modifications at TSS and TTS of genes with different transcription levels plotted separately. **Figure S4**. Interpretation of a CART tree. **Table S1.** Sources of gene expression and histone modifications data of MSC, hESC-h1, and IMR-90 cell lines. **Table S2.** Correspondence between Active/Inactive classification of genes in CD4+ T-cells based on empirical data and CART-based bioinformatics approach of present study (five node tree of Fig. 4c). **Table S3.** Gene activity prediction frequencies for IMR90, hESC-h1, and MSC cells based on five node CART trees for each of the histone modifications on the 24 nucleosome sized regions. (DOC 1328 kb)

## Abbreviations

CART: Classification and regression tree
CD4: Cluster of differentiation 4
ChIP-Seq: Chromatin immunoprecipitation-sequencing protocol
MDR: Multifactorial dimensionality reduction
RMA: Robust multi-array average
TSS: Transcription start site
TTS: Transcription termination site

## References

[1] Kornberg RD (1974). Chromatin structure: a repeating unit of histones and DNA. Science.

[2] Kornberg RD, Thomas JO (1974). Chromatin structure; oligomers of the histones. Science.

[3] Natsume-Kitatani Y, Shiga M, Mamitsuka H (2011). Genome-wide integration on transcription factors, histone acetylation and gene expression reveals genes co-regulated by histone modification patterns. PLoS One.

[4] Nair NU, Lin Y, Manasovska A, Antic J, Grnarova P, Sahu AD (2014). Study of cell differentiation by phylogenetic analysis using histone modification data. BMC Bioinformatics.

[5] Strahl BD, Allis CD (2000). The language of covalent histone modifications. Nature.

[6] Bannister AJ, Kouzarides T (2011). Regulation of chromatin by histone modifications. Cell Res.

[7] Greer EL, Shi Y (2012). Histone methylation: a dynamic mark in health, disease and inheritance. Nat Rev Genet.

[8] Yun M, Wu J, Workman JL, Li B (2011). Readers of histone modifications. Cell Res.

[9] Zhang Y, Reinberg D (2001). Transcription regulation by histone methylation: interplay between different covalent modifications of the core histone tails. Genes Dev.

[10] Dreveny I, Deeves SE, Fulton J, Yue B, Messmer M, Bhattacharya A (2014). The double PHD finger domain of MOZ/MYST3 induces alpha-helical structure of the histone H3 tail to facilitate acetylation and methylation sampling and modification. Nucleic Acids Res.

[11] Kouzarides T (2007). Chromatin modifications and their function. Cell.

[12] Waters R, van Eijk P, Reed S (2015). Histone modification and chromatin remodeling during NER. DNA repair.

[13] Cosgrove MS (2012). Writers and readers: deconvoluting the harmonic complexity of the histone code. Nat Struct Mol Biol.

[14] Rusk N (2012). Writing the histone code. Nat Methods.

[15] Chen H, Lonardi S, Zheng J (2014). Deciphering histone code of transcriptional regulation in malaria parasites by large-scale data mining. Comput Biol Chem.

[16] Turner BM (2000). Histone acetylation and an epigenetic code. BioEssays.

[17] Fillingham J, Greenblatt JF (2008). A histone code for chromatin assembly. Cell.

[18] Heintzman ND, Hon GC, Hawkins RD, Kheradpour P, Stark A, Harp LF (2009). Histone modifications at human enhancers reflect global cell-type-specific gene expression. Nature.

[19] Huang RH, Huang J, Cathcart H, Smith S, Poduslo SE (2007). Genetic variants in brain-derived neurotrophic factor associated with Alzheimer’s disease. J Med Genet.

[20] Ingoldsby HW, Webber M. Wall, D. Scarrott, C. Newell, J. Callagy, G. Prediction of Oncotype DX and TAILORx risk categories using histopathological and immunohistochemical markers by classification and regression tree (CART) analysis. Breast. 2013. 10.1016/j.breast.2013.04.008 23643806

[21] Moore JH (2010). Detecting, characterizing, and interpreting nonlinear gene-gene interactions using multifactor dimensionality reduction. Adv Genet.

[22] Karlic RC, Chung HR, Lasserre J, Vlahovicek K, Vingron M. Histone modification levels are predictive for gene expression. Proc Natl Acad Sci U S A. 2010;107(7):2926–31. 10.1073/pnas.0909344107 PMC2814872 20133639

[23] Hsieh AR, Hsiao CL, Chang SW, Wang HM, Fann CS (2011). On the use of multifactor dimensionality reduction (MDR) and classification and regression tree (CART) to identify haplotype-haplotype interactions in genetic studies. Genomics.

[24] Ritchie MD, Hahn LW, Roodi N, Bailey LR, Dupont WD, Parl FF (2001). Multifactor-dimensionality reduction reveals high-order interactions among estrogen-metabolism genes in sporadic breast cancer. Am J Hum Genet.

[25] Hahn LW, Ritchie MD, Moore JH (2003). Multifactor dimensionality reduction software for detecting gene-gene and gene-environment interactions. Bioinformatics.

[26] Wang Z, Zang C, Rosenfeld JA, Schones DE, Barski A, Cuddapah S (2008). Combinatorial patterns of histone acetylations and methylations in the human genome. Nat Genet.

[27] Barski A, Cuddapah S, Cui K, Roh TY, Schones DE, Wang Z (2007). High-resolution profiling of histone methylations in the human genome. Cell.

[28] Zhang Y, Lv J, Liu H, Zhu J, Su J, Wu Q (2010). HHMD: the human histone modification database. Nucleic Acids Res.

[29] Wang Z, Zang C, Cui K, Schones DE, Barski A, Peng W (2009). Genome-wide mapping of HATs and HDACs reveals distinct functions in active and inactive genes. Cell.

[30] Sedaghat AR, German J, Teslovich TM, Cofrancesco J, Jie CC, Talbot CC (2008). Chronic CD4+ T-cell activation and depletion in human immunodeficiency virus type 1 infection: type I interferon-mediated disruption of T-cell dynamics. J Virol.

[31] Li G, Zhang W, Zeng H, Chen L, Wang W, Liu J (2009). An integrative multi-platform analysis for discovering biomarkers of osteosarcoma. BMC Cancer.

[32] Ravoet M, Sibille C, Gu C, Libin M, Haibe-Kains B, Sotiriou C (2009). Molecular profiling of CD3-CD4+ T cells from patients with the lymphocytic variant of hypereosinophilic syndrome reveals targeting of growth control pathways. Blood.

[33] Annibali V, Ristori G, Angelini DF, Serafini B, Mechelli R, Cannoni S (2011). CD161(high)CD8+T cells bear pathogenetic potential in multiple sclerosis. Brain.

[34] McLaren PJ, Ball TB, Wachihi C, Jaoko W, Kelvin DJ, Danesh A (2010). HIV-exposed seronegative commercial sex workers show a quiescent phenotype in the CD4+ T cell compartment and reduced expression of HIV-dependent host factors. J Infect Dis.

[35] Mold JE, Venkatasubrahmanyam S, Burt TD, Michaelsson J, Rivera JM, Galkina SA (2010). Fetal and adult hematopoietic stem cells give rise to distinct T cell lineages in humans. Science.

[36] Le Dieu R, Taussig DC, Ramsay AG, Mitter R, Miraki-Moud F, Fatah R (2009). Peripheral blood T cells in acute myeloid leukemia (AML) patients at diagnosis have abnormal phenotype and genotype and form defective immune synapses with AML blasts. Blood.

[37] Piccaluga PP, Agostinelli C, Califano A, Rossi M, Basso K, Zupo S (2007). Gene expression analysis of peripheral T cell lymphoma, unspecified, reveals distinct profiles and new potential therapeutic targets. J Clin Invest.

[38] Bonacci B, Edwards B, Jia S, Williams CB, Hessner MJ, Gauld SB (2012). Requirements for growth and IL-10 expression of highly purified human T regulatory cells. J Clin Immunol.

[39] Allantaz F, Cheng DT, Bergauer T, Ravindran P, Rossier MF, Ebeling M (2012). Expression profiling of human immune cell subsets identifies miRNA-mRNA regulatory relationships correlated with cell type specific expression. PLoS One.

[40] Tsitsiou E, Williams AE, Moschos SA, Patel K, Rossios C, Jiang X (2012). Transcriptome analysis shows activation of circulating CD8+ T cells in patients with severe asthma. J Allergy Clin Immunol.

[41] Gillies CE, Siadat MR, Patel NV, Wilson GD (2013). A simulation to analyze feature selection methods utilizing gene ontology for gene expression classification. J Biomed Inform.

[42] Moore JH (2004). Computational analysis of gene-gene interactions using multifactor dimensionality reduction. Expert Rev Mol Diagn.

[43] Chang CW, Cheng WC, Chen CR, Shu WY, Tsai ML, Huang CL (2011). Identification of human housekeeping genes and tissue-selective genes by microarray meta-analysis. PLoS One.

[44] Noble WS (2006). What is a support vector machine?. Nat Biotechnol.

[45] Ernst J, Kellis M (2012). ChromHMM: automating chromatin-state discovery and characterization. Nat Methods.

[46] Abell AN, Jordan NV, Huang W, Prat A, Midland AA, Johnson NL (2011). MAP3K4/CBP-regulated H2B acetylation controls epithelial-mesenchymal transition in trophoblast stem cells. Cell Stem Cell.

[47] Henikoff S, Shilatifard A (2011). Histone modification: cause or cog?. Trends Genet.

[48] Shahbazian MD, Grunstein M (2007). Functions of site-specific histone acetylation and deacetylation. Annu Rev Biochem.

[49] Li B, Carey M, Workman JL (2007). The role of chromatin during transcription. Cell.

