# Supplementary material for: Confident gene activity prediction based on single histone modification H2BK5ac in human cell lines
Source: BMC Bioinformatics. 2017 Jan 25;18:67. doi: 10.1186/s12859-016-1418-6 (PMC5264486; doi:10.1186/s12859-016-1418-6)
Supplement: Additional file 1: Figure S1. — shows five localities that together are expected to encompass 24 gene regions. Figure S2. Flow chart of analyses performed with CART. Figure S3. Occurrence frequencies of individual histone modifications at TSS and TTS of genes with different transcription levels plotted separately. Figure S4. Interpretation of a CART tree. Table S1. Sources of gene expression and histone modifications data of MSC, hESC-h1, and IMR-90 cell lines. Table S2. Correspondence between Active/Inactive classification of genes in CD4+ T-cells based on empirical data and CART-based bioinformatics approach of present study (five node tree of Fig. 4c). Table S3. Gene activity prediction frequencies for IMR90, hESC-h1, and MSC cells based on five node CART trees for each of the histone modifications on the 24 nucleosome sized regions. (DOC 1328 kb) [file 12859_2016_1418_MOESM1_ESM.doc]

**
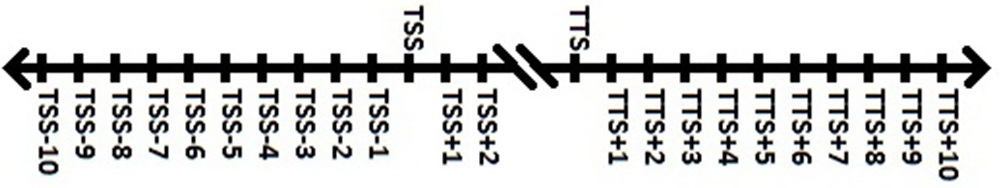
**

**Figure S1. Schematic representation of five localities wherein histone modifications at 24 gene regions were analyzed.** The five regions included the transcription start site (TSS; one gene region), 2000 bp upstream of the TSS (TSS-1–TSS-10; 10 gene regions), 400 bp downstream of the TSS (TSS+1 and TSS+2; two gene regions), the transcription termination site (TTS; one gene region), and 2000 bp downstream of the TTS (TTS+1–TTS+10; 10 gene regions). Each gene region encompasses approximately 200 nucleotides.


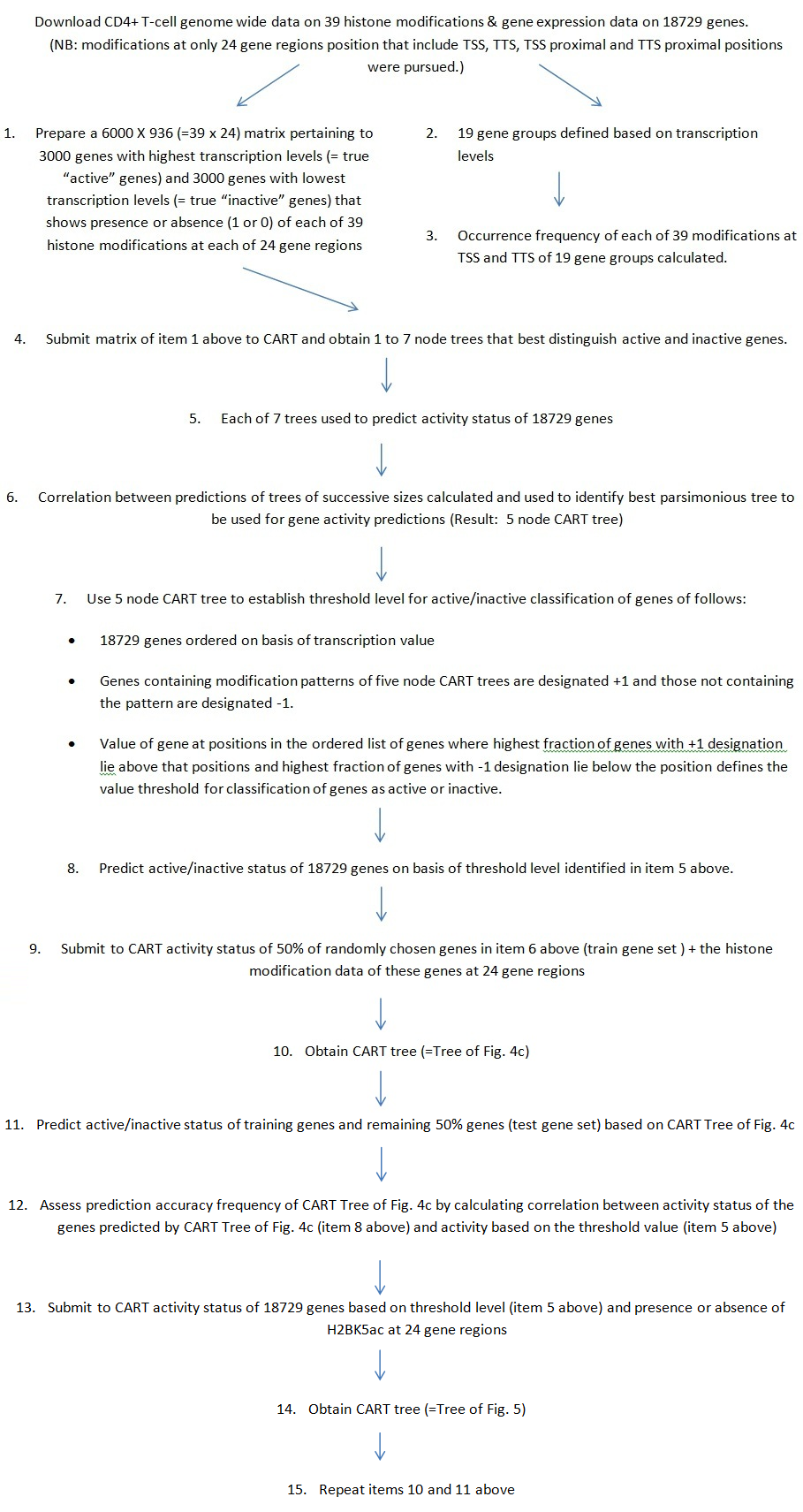


**Figure S2-** **Flow chart of analyses performed with CART***

*Analysis done on 39 modifications alone at each of 24 gene regions separately (Fig. 3) not included in flow chart. A text summary of what we did is as follows: Five node tree that signaled the importance of H2BK5ac was originally derived using data of the 3,000 genes with highest transcription signals and the 3,000 genes with the lowest signals (Figure. 2b). All data pertaining to gene expression signals were directly adopted from the source literatures used. The five node tree was subsequently used to establish a threshold signal level for predicting activity status of all 18792 genes as described in section entitled “Establishment of criterion for assessment of active genes and classification of the 18792 genes as active or inactive”. Then, a tree based on status of all 39 modifications at all 24 gene regions pertaining to half of the 18792 genes (the training genes) was derived that again showed the importance of the H2BK5ac modification (Figure 4c). The predictions of activity status of all 18792 genes using trees of Figures 2b and 4c showed high correspondence. Finally, a tree based only on status of H2BK5ac (Figure 5) showed equally high activity prediction accuracy for the 18792 genes.


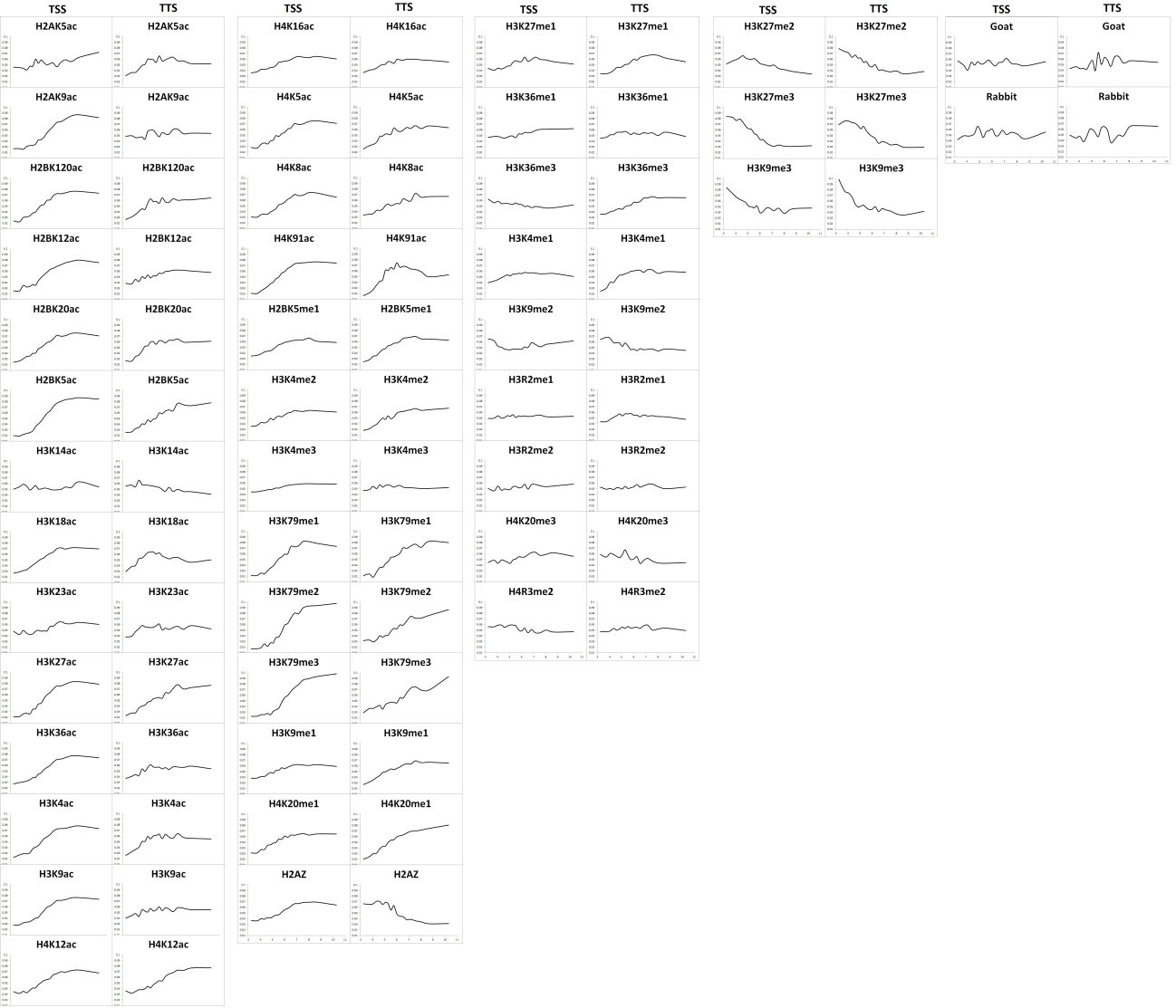


**Figure S3. Occurrence frequencies of individual histone modifications at TSS and TTS of genes with different transcription levels plotted separately.** Results of the various modifications at TSS and at TTS are shown independently for sake of clarity. The values of the occurrence frequencies of modifications (Y-axis) of all the plots range from 0.01- 0.1. The x-axis of all the plots represent log2 averaged normalized transcription values of the 19 gene groups. Data on control nonspecific goat and rabbit antibodies are also presented.


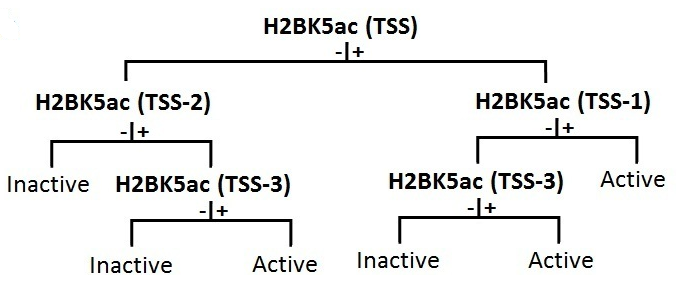


**Figure S4. Interpretation of a CART tree**. This example tree predicts that If H2BK5ac is present at positions TSS and TSS-1 (right branch of tree), at TSS and TSS-3 (right branch of tree), or at TSS-2 and TSS-3 (left branch of tree), the gene will be active. The tree predicts that a gene with other modification patterns will be inactive.

| **Table S1. Sources of gene expression and histone modifications data of MSC, hESC-h1, and IMR-90 cell lines** | | |
| --- | --- | --- |
| **Cell line** | **Expression data source*** | **Investigated modifications**** |
| MSC | GSM139881  GSM139882  GSM139883  GSM139888  GSM139889  GSM139890 | H3K36me3, H3K4me1, H3K9me3, H3K27ac, H3K27me3, H3K4me3, H2A.Z, H2AK5ac, H2BK12ac, H2BK5ac, H3K14ac, H3K18ac, H3K23ac, H3K4ac, H3K4me2, H3K79me1, H3K9ac, H4K8ac, H4K91ac, H2BK120ac, H2BK14ac, |
|  |  |  |
| hESC-h1 | GSM1341467  GSM1341468  GSM225042  GSM604824  GSM604828 | H2AK5ac, H2BK120ac, H2BK12ac, H2BK15ac, H2BK20ac, H2BK5ac, H3K14ac, H3K18ac, H3K23ac, H3K23me2, H3K27ac, H3K27me3, H3K36me3, H3K4ac, H3K4me1, H3K4me2, H3K4me3, H3K56ac, H3K79me1, H3K79me2, H3K9ac, H3K9me3, H4K20me1, H4K5ac, H4K91ac |
|  |  |  |
| IMR-90 | GSM438363 | H2A.Z, H2AK5ac, H2AK9ac, H2BK120ac, H2BK12ac, H2BK15ac, H2BK20ac, H2BK5ac, H3K14ac, H3K18ac, H3K23ac, H3K27ac, H3K27me3, H3K36me3, H3K4ac, H3K4me1, H3K4me2, H3K4me3, H3K56ac, H3K79me1, H3K79me2, H3K9ac, H3K9me1, H3K9me3, H4K20me1, H4K5ac, H4K5ac, H4K8ac, H4K91ac, |

*http://www.ncbi.nlm.nih.gov/geo/,

**http://www.ncbi.nlm.nih.gov/geo/(GSE16256)

| **Table S2. Correspondence between Active/Inactive classification of genes in CD4+ T-cells based on empirical data and CART-based bioinformatics approach of present study (five node tree of Fig. 4c).** | | | | |
| --- | --- | --- | --- | --- |
| **Empirical data*/ Threshold data** | **A/A** | **A/I** | **I/A** | **I/I** |
|  | 1983 | 61 | 356 | 1747 |

*Empirical data derived from reference 26 (Supplementary tables ts1 and ts3 in the reference), wherein authors stated that a cutoff expression value of 200 can be used for identification of expressed genes. Data on 4147 genes was available in both reference 26 and in our CART analysis. The distribution of genes designated active (A) or inactive (I) in both studies, or active in one and inactive in the other is presented. Of the 4147 genes, the empirical and bioinformatics approach designated the same activity status for 3730 (89.4%) genes.

**Table S3. Gene activity prediction frequencies for IMR90, hESC-h1, and MSC cells based on five node CART trees for each of the** histone modifications on the 24 nucleosome sized regions.

| **Histone modification** | H3K27ac | H2BK5ac | H3K9ac | H3K79me1 | H4K8ac | H3K4me3 | H3K56ac | H3K79me2 | H3K4ac | H3K36me3 |
| --- | --- | --- | --- | --- | --- | --- | --- | --- | --- | --- |
| **Prediction accuracy** | 0.782 | 0.781 | 0.777 | 0.775 | 0.775 | 0.773 | 0.769 | 0.768 | 0.767 | 0.764 |
| **Histone modification** | H4K91ac | H3K18ac | H2BK12ac | H3K14ac | H4K5ac | H3K4me2 | H2BK15ac | H2AK9ac | H3K23ac | H2BK120ac |
| **Prediction accuracy** | 0.759 | 0.756 | 0.754 | 0.754 | 0.753 | 0.748 | 0.748 | 0.747 | 0.740 | 0.738 |
| **Histone modification** | H2BK20ac | H2AZ | H4K20me1 | H3K4me1 | H3K9me1 | H3k27me3 | H3K9me3 | H2AK5ac |  |  |
| **Prediction accuracy** | 0.733 | 0.721 | 0.719 | 0.714 | 0.666 | 0.611 | 0.599 | 0.448 |  |  |

IMR-90

| **Histone modification** | H3K27ac | H4K5ac | H3K4me3 | H3K9ac | H2BK5ac | H3K4me2 | H3K56ac | H4K20me1 | H3K18ac |
| --- | --- | --- | --- | --- | --- | --- | --- | --- | --- |
| **Prediction accuracy** | 0.729 | 0.727 | 0.716 | 0.704 | 0.700 | 0.693 | 0.674 | 0.662 | 0.658 |
| **Histone modification** | H4K91ac | H3k27me1 | H3K4ac | H2BK120ac | H3K14ac | H3K79me1 | H3K4me1 | H2BK12ac | H3K23me2 |
| **Prediction accuracy** | 0.650 | 0.650 | 0.644 | 0.640 | 0.634 | 0.632 | 0.631 | 0.629 | 0.627 |
| **Histone modification** | H3K23ac | H2BK15ac | H2AK5ac | H3K79me2 | H2BK20ac | H3K9me3 | H3K36me3 |  |  |
| **Prediction accuracy** | 0.616 | 0.615 | 0.614 | 0.600 | 0.598 | 0.593 | 0.471 |  |  |

**hESC-h1**

| **Histone modification** | H3K27me3 | H3K9ac | H3K36me3 | H2BK5ac | H3K18ac | H3K14ac | H3K4me1 | H2AZ |
| --- | --- | --- | --- | --- | --- | --- | --- | --- |
| **Prediction accuracy** | 0.664 | 0.641 | 0.639 | 0.639 | 0.635 | 0.631 | 0.622 | 0.619 |
| **Histone modification** | H3K23ac | H4K91ac | H2BK12ac | H3K4me3 | H3K4ac | H3K79me1 | H4K8ac | H2BK120ac |
| **Prediction accuracy** | 0.617 | 0.613 | 0.610 | 0.609 | 0.609 | 0.609 | 0.609 | 0.608 |
| **Histone modification** | H2AK5ac | H3K27ac | H3K4me2 | H3K9me3 |  |  |  |  |
| **Prediction accuracy** | 0.601 | 0.594 | 0.591 | 0.571 |  |  |  |  |

**MSC**
